# Preparation of a Bis-GMA-Free Dental Resin System with Synthesized Fluorinated Dimethacrylate Monomers

**DOI:** 10.3390/ijms17122014

**Published:** 2016-12-01

**Authors:** Shuzhen Luo, Wenbin Zhu, Fang Liu, Jingwei He

**Affiliations:** College of Materials Science and Engineering, South China University of Technology, Guangzhou 510641, China; top_silly@sina.cn (S.L.); zhu_wb@outlook.com (W.Z.); msfliu@126.com (F.L.)

**Keywords:** dental resin, Bis-GMA free, fluorinated dimethacrylate, physicochemical properties

## Abstract

With the aim of reducing human exposure to Bisphenol A (BPA) derivatives in dentistry, a fluorinated dimethacrylate monomer was synthesized to replace 2,2-bis[4-(2-hydroxy-3-methacryloy-loxypropyl)-phenyl]propane (Bis-GMA) as the base monomer of dental resin. After mixing with reactive diluent triethyleneglycol dimethacrylate (TEGDMA), fluorinated dimethacrylate (FDMA)/TEGDMA was prepared and compared with Bis-GMA/TEGDMA in physicochemical properties, such as double bond conversion (DC), volumetric shrinkage (VS), water sorption (WS) and solubility (WSL), flexural strength (FS) and modulus (FM). The results showed that, when compared with Bis-GMA based resin, FDMA-based resin had several advantages, such as higher DC, lower VS, lower WS, and higher FS after water immersion. All of these revealed that FDMA had potential to be used as a substitute for Bis-GMA. Of course, many more studies, such as biocompatibility testing, should be undertaken to prove whether FDMA could be applied in clinic.

## 1. Introduction

Since they were first applied in dentistry in the 1960s, light-curable methacrylate-based dental composites have been widely used in clinic because of their advantages, such as excellent aesthetic property and easy handling [1]. Usually, dental composites consist of a methacrylate-based resin matrix, a photoinitiation system, and silane coupling agent-treated fillers [2]. The commonly used resin matrix is a mixture of two or more dimethacrylate monomers chosen from 2,2-bis[4-(2-hydroxy-3-methacryloy-loxypropyl)-phenyl]propane (Bis-GMA), ethoxylated Bis-GMA (Bis-EMA), 1,6-bis-[2-methacryloloxyethoxycarbonyl-amino]-2,4,4-trimethyl-hexane (UDMA), and triethyleneglycol dimethacrylate (TEGDMA) [2,3,4]. The performances of dental composites were strongly influenced by the composition of the resin matrix [5,6,7].

Bis-GMA is a predominant monomer used in commercial dental composites, and the dominance of Bis-GMA is attributed to its low volumetric shrinkage, high reactivity, good mechanical properties, low volatility and diffusivity into tissues [2,8]. However, the estrogenic potential problem of Bis-GMA-based dental composites has come out, because Bis-GMA was synthesized from bisphenol A (BPA) and glycidyl methacrylate (GMA), and BPA is one kind of endocrine disrupting compound that can cause several health problems, such as male reproductive abnormalities [9,10], spermatogenesis impairment [11,12], and high probability of heart disease and diabetes [13,14]. Though it was reported that Bis-GMA could not hydrolyze into BPA [15,16,17], BPA was still detected to be released out of commercial Bis-GMA based dental composites [8,18]. With the aim of minimizing human exposure to BPA, using methacrylate monomers that are not derived from BPA might be an effective way.

In order to prepare Bis-GMA-free dental materials, UDMA, which is another typical dimethacrylate monomer applied in dentistry, has been considered to be used to replace Bis-GMA as the base resin of dental materials [19]. Unfortunately, compared with Bis-GMA-based resin, UDMA-based resin had a critical shortcoming in its higher volumetric shrinkage [20]. The higher volumetric shrinkage of UDMA-based resin might lead to a greater marginal gap between tooth and restorations, resulting in a higher possibility of secondary caries [21]. Liang and coworkers synthesized a siloxane containing BPA-free dimethacrylate monomer (SiMA) and applied it into dental resin. Compared to Bis-GMA based resin, SiMA had advantages such as higher double bond conversion, lower volumetric shrinkage, and lower water sorption, but SiMA-based resin had much lower flexural strength and modulus [22,23].

Fluorocarbon-containing polymers were reported to have several advantages such as high hydrophobicity, excellent resistance to a wide range of chemicals, potential resistance to microbial attachment, and good biocompatibility [2]. In addition, several researches showed that mechanical properties could be improved by introducing fluorine into a dental monomer [24,25,26]. All of these properties make fluorinated polymers very attractive for dental application.

In this research, a new dimethacrylate monomer with fluorine (FDMA) was synthesized and applied to dental resin with the aim of preparing Bis-GMA free dental resin. An unfilled dental resin was prepared by mixing FDMA and TEGDMA together. Properties such as viscosity, double bond conversion, volumetric shrinkage, water sorption and solubility, flexural strength and modulus of FDM- based dental resin were investigated and compared with Bis-GMA-based dental resin.

## 2. Results

**Figure 1:**
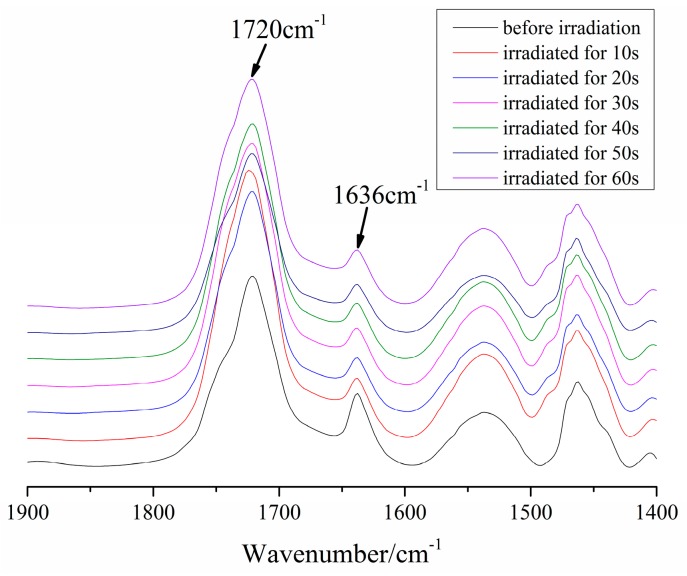
FT-IR spectra during the irradiation process: taking FDMA-based resin as an example.

**Figure 2:**
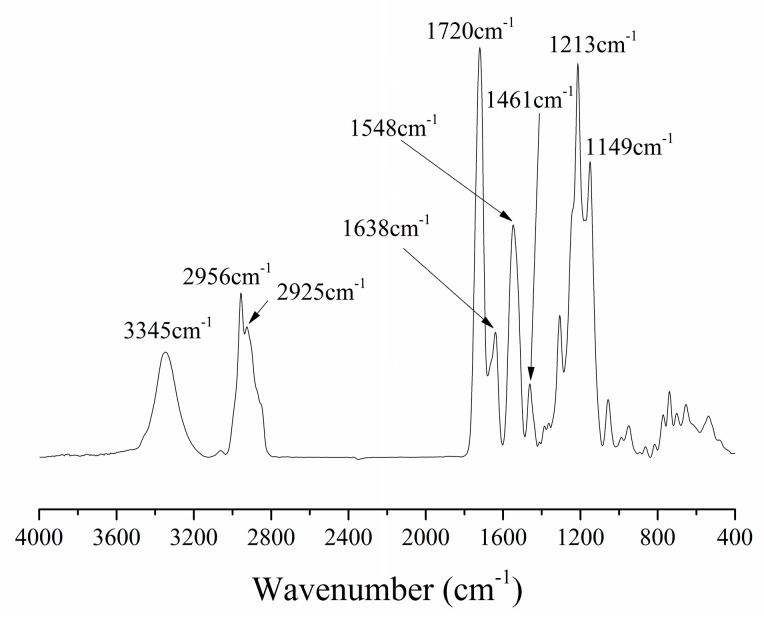
Fourier transform infrared spectroscopy (FT-IR) spectrum of fluorinated dimethacrylate (FDMA).

FT-IR and ^1^H-NMR spectra of FDMA are shown in Figure 1 and Figure 2, respectively. Figure 1 and Figure 2 revealed that the structure of synthesized monomer was the same as designed monomer.

**Figure 3:**
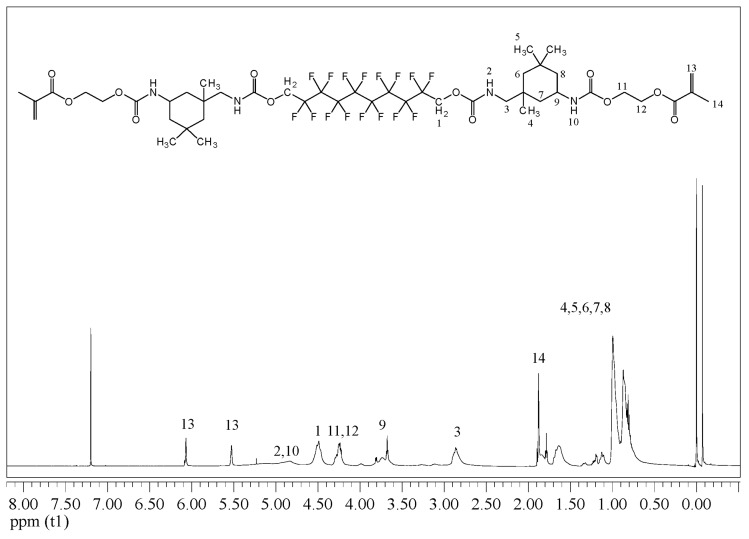
Proton Nuclear Magnetic Resonance (^1^H-NMR) spectrum of FDMA.

The viscosity of each resin system was listed in Table 1; the FDMA-based resin system had higher viscosity than the Bis-GMA-based resin system (*p* < 0.05). Figure 3 depicts the curves of irradiation time versus the double conversion (DC) of each resin system, and DCs at the irradiation time of 60 s were shown in Table 1. From Figure 3 and Table 1, it could be seen that the FDMA-based resin system had a higher photopolymerization rate in the early stage of polymerization and higher DC (*p* < 0.05) than the Bis-GMA-based resin system. The results of volumetric shrinkage (VS) were also shown in Table 1, and the FDMA-based resin system had lower VS than the Bis-GMA-based resin system (*p* < 0.05).

The results of water sorption (WS), water solubility (WSL), flexural strength (FS) and flexural modulus (FM) before and after water immersion were summarized in Table 2. Compared with the Bis-GMA-based polymer, the FDMA-based polymer had lower WS (*p* < 0.05) and higher WSL (*p* < 0.05). Before water immersion, the FDMA-based polymer had comparable FS and FM as the Bis-GMA-based polymer (*p* > 0.05). After water immersion, the FS and FM of every polymer decreased significantly (*p* < 0.05); the FS of the FDMA-based polymer became higher than that of the Bis-GMA-based polymer (*p* < 0.05), and the FM of the FDMA-based polymer was still the same as the FM of the Bis-GMA-based polymer (*p* > 0.05).

## 3. Discussion

The resin matrix used in dental composites should fulfill a series of requirements in physicochemical properties, such as high photopolymerization rate, low volumetric shrinkage, sufficient mechanical properties, and low water sorption [2]. In order to prepare the Bis-GMA-free dental resin system, the new resin system should have comparable or better physicochemical properties. In this research, the differences in properties between Bis-GMA-based resin and FDMA-based resin was mainly attributed to the difference in structure between Bis-GMA and FDMA.

The viscosity of monomer, defined as a parameter that reflects the resistance of molecules to flow, has been reported to influence the photopolymerization rate and DC [27,28]. At an early stage of polymerization, an insoluble cross-linking network will form when polymerizing dimethacrylate monomers and the termination step of polymerization will change from chemical controlled to diffusion controlled. This variance will lead to a decrease in the termination rate and an increase in the polymerization rate which is known as autoacceleration or the gel effect, and this effect is more pronounced in highly viscous monomers [28]. Therefore, the higher initial polymerization rate of FDMA-based resin should be due to its higher viscosity. On the other hand, high viscosity that is induced by the intermolecular interaction is not good for the dental resin system, because it can decrease the flexibility of the polymer chain and the mobility of the reactive monomer, leading to a decrease in DC [28,29,30]. However, FDMA-based resin had higher viscosity than Bis-GMA-based resin, but it still had higher DC in this work. It might be attributed to the -NH- groups in FDMA, which can increase the mobility of radical sites on the network by causing a chain transfer reaction [28]. This phenomenon was also observed in previous research, i.e., UDMA had higher DC than ethoxylated bisphenol-A-dimethacrylate (Bis-EMA), even though UDMA had higher viscosity than Bis-EMA [28].

As an inevitable drawback of methacrylate-based dental composite, volumetric shrinkage should be reduced as much as possible. According to abundant studies, volumetric shrinkage was revealed to be dependent on double bond concentration and conversion [23,31,32,33,34,35,36,37]. Lower double bond concentration and conversion will lead to lower volumetric shrinkage. Though FDMA-based resin had much higher DC than Bis-GMA-based resin, its VS was lower than that of Bis-GMA-based resin. This should be mainly attributed to the higher molecular weight of FDMA (1166), which made the double bond concentration of FDMA (8.58 × 10^−4^ mol/g) significantly lower than that of Bis-GMA (1.95 × 10^−3^ mol/g).

In the structure of FDMA, there exist four flexible urethane linkages, which might make the FDMA-based polymer chain more flexible than the Bis-GMA-based polymer chain. However, the FS and FM of the FDMA-based polymer were comparable with those of the Bis-GMA-based polymer; this might be due to the higher cross-linking density of FDMA-based resin caused by its higher DC. The same result was also found in other studies comparing urethane-based resins and Bis-GMA-based resin; urethane-based resins had FS and FM comparable to those of Bis-GMA-based resin because of their higher cross-linking density caused by the higher DC [4,20]. In addition, though FDMA has a long -(CF_2_)_8_- chain, it still performed as a rigid compound because the rotation of the -(CF_2_)_8_- chain is bonded by bulky fluorine [26]. This should be another reason why the FDMA-based polymer had FS and FM comparable to those of the Bis-GMA based polymer.

The WS of dental materials influences the long-term stability of dental materials appliances in an aqueous environment, such as in the mouth, because the water intrusion can induce them several adverse effects, such as hydrolysis of the polymeric network [38], reducing thermal stability [39], impairing mechanical properties [40], and elution of unreacted monomers [41]. Previous research has reported that fluorinated resins demonstrated excellent hydrophobicity, which was much higher than that of Bis-GMA-based resin, and water uptake of fluorinated resins was much lower than that of Bis-GMA-based resin [42]. Therefore, the lower WS of FDMA-based resin might be due to the fluorinated structure in FDMA. Because of its lower WS, FDMA-based resin might have better water resistance than Bis-GMA-based resin. It could be observed clearly that, before water immersion, FDMA and Bis-GMA-based resins had comparable FS, while FS of FDMA became higher than that of Bis-GMA-based resin after water immersion, and the lower descent rate (8.2% for FDMA based resin and 22.6% for Bis-GMA based resin) of FS for FDMA-based resin after water immersion could be one sign of its better water resistance.

The WSL reveals the amount of unreacted monomers being leached out of polymeric networks. The release of monomers is the main source of cytotoxicity and tissue inflammation [43]. It was reported that WSL was correlated with DC, and higher DC could lead to lower WSL [44]. However, compared with Bis-GMA-based resin, FDMA-based resin had higher WSL, though it had higher DC. The same phenomenon was also observed in some other studies, i.e., some urethane methacrylate based resin systems had higher WSL than the Bis-GMA-based resin system, even though their DCs were higher [3,45]. This should be attributed to the leachability of unreacted monomers in the polymeric network. The inter-molecular hydrogen bonds formed by -OH groups were stronger than hydrogen bonds formed by -NH- because of the higher cohesive energy density of -OH groups [44]. Therefore, the unreacted monomer could be absorbed to the surrounding network much more tightly in the Bis-GMA-based polymer as well as being harder to leach out of the polymer, leading to lower WSL of the Bis-GMA-based polymer.

## 4. Materials and Methods

### 4.1. Materials

Isophorone diisocyanate (IPDI), 1*H*,1*H*,10*H*,10*H*-Perfluorodecane-1,10-diol (PFDOL), 2-hydroxyethyl methacrylate (HEMA), TEGDMA, and extra dry tetrahydrofuran (THF) were purchased from J & K Scientific Ltd. (Guangzhou, China). Camphorquinone (CQ), and dibutyltin dilaurate (DBTDL) were purchased from Tokyo Chemical Industry Co. (Tokoy, Japan). Bis-GMA, and 2-(*N*,*N*-dimethylamino)ethyl methacrylate (DMAEMA) were purchased from Sigma-Aldrich Co. (St. Luois, MO, USA). All of the reagents were used directly without purification.

### 4.2. Synthesis of FDMA

**Scheme 1:**
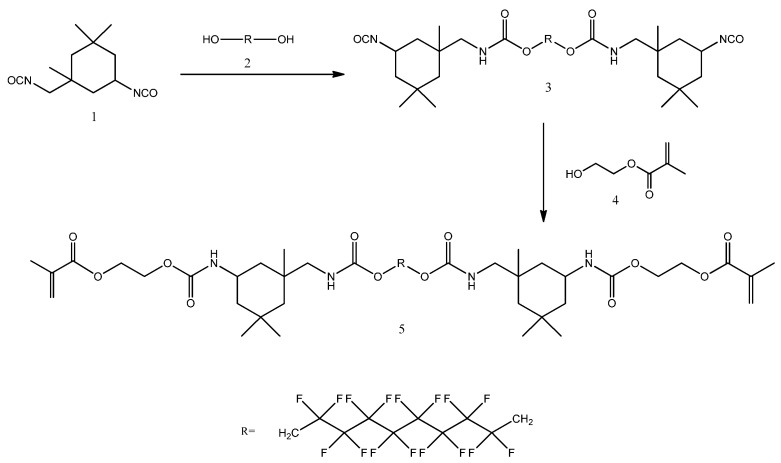
Synthesis route of FDMA.

FDMA was synthesized according to the reaction route shown in Scheme 1. Firstly, a urethane precursor was synthesized through the reaction between (22.23 g, 0.10 mol) IPDI (**1**) and (23.11 g, 0.05 mol) PFDOL (**2**) at 45 °C. In this reaction, THF and a few droplets of DBTDL were used as the solvent and catalyst, respectively. When the -NCO groups content reached half of the initial content (determined by dibutyl amine titration) to form (**3**), 13.01 g (0.10 mol) of HEMA (**4**) were added into the reactor and the reaction was continued under 45 °C. The reaction was stopped until the infrared absorbance peak of the -NCO group (2270 cm^−1^) disappeared in the FT-IR (Fourier Transform Infrared; Vector33, Bruker Co., Bremen, Germany) spectra of the samples taken from the reaction medium every 1 h. After removing the THF by distillation under vacuum, the product FDMA (**5**) was obtained as a colorless viscose liquid. The structure of FDMA was investigated by FT-IR and ^1^H-NMR (Proton Nuclear Magnetic Resonance Instrument; Avance AV 400 MHz, Bruker Co., Fällanden, Switzerland) spectra.

### 4.3. Preparation of Resin Formulation

FDMA (or Bis-GMA), TEGDMA, CQ, and DMAEMA were mixed together to form an unfilled dental resin system at a mass ratio of 49.3:49.3:0.7:0.7 (FDMA (or Bis-GMA):TEGDMA:CQ:DMAEMA). CQ and DMAEMA were used as a photoinitiation system. All of the compounds were mixed and stirred magnetically at room temperature until a uniform system was obtained. The prepared dental resin systems were stored in the dark before being used.

### 4.4. Measurement of Viscosity of Dental Resin

The viscosities of the prepared dental resin systems were measured by a rotary viscometer (NDJ-79, Shanghai Pingxuan Instrument Co., Ltd., Shanghai, China) with a plate diameter of 1.6 cm. An amount of 18 mL dental resin was used for each test. The measurement was taken at 25 °C with a rotor speed of 750 r/min. Measurement was repeated four times for each dental resin system.

### 4.5. Measurement of Double Bond Conversion

**Figure 4:**
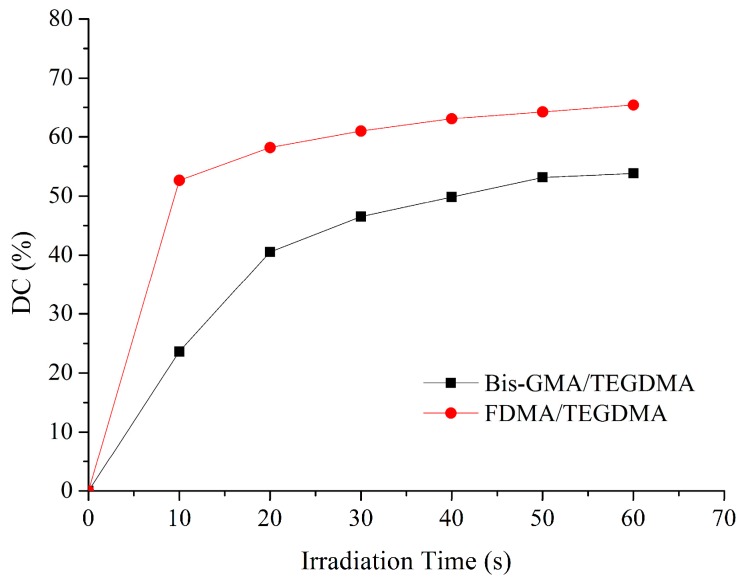
The curves of double conversion (DC) versus irradiation time of each dental resin system. Bis-GMA: 2,2-bis[4-(2-hydroxy-3-methacryloy-loxypropyl)-phenyl]propane; TEGDMA: triethyleneglycol dimethacrylate; FDMA: fluorinated dimethacrylate.

The DCs at different irradiation times were monitored by a FT-IR according to the method reported previously by Luo et al. [45]. The resin sample was coated on a KBr Pellets gently to form a very thin film and the absorbance peak of the uncured sample was obtained. Then, a dental light source (Mini LED Curing Lights, λ = 390–510 nm, I ≈ 1250 mW·cm^−2^, 8 mm of curing light diameter, Satelec Inc., Bordeaux, France) was used to irradiate the resin sample at room temperature. The spectra during the irradiation process was recorded every 10 s for 1 min (as shown in Figure 4). The DC was calculated from the aliphatic C=C peak at 1636 cm^−1^ and normalized against the carbonyl C=O peak at 1720 cm^−1^ according to the Equation (1):

(1) $$\text{DC}(t)=\frac{{\left(A_{C=C}/A_{C=O}\right)}_{0}-{\left(A_{C=C}/A_{C=O}\right)}_{t}}{{\left(A_{C=C}/A_{C=O}\right)}_{0}}$$

where A_C=C_ and A_C=O_ are the absorbance peak area of methacrylate C=C at 1636 cm^−1^ and carbonyl at 1720 cm^−1^, respectively; (A_C=C_/A_C=O_)_0_ and (A_C=C_/A_C=O_)_t_ are the normalized absorbance of the functional group at the radiation time of 0 and t, respectively; DC(t) is the conversion of methacrylate C=C as a function of radiation time.

### 4.6. Measurement of Volumetric Shrinkage

The VS of dental resin was investigated through the variation in density before and after photopolymerization, and the density was determined according to the Archimedes’ principle. The measurement was carried out with a commercial Density Determination Kit of the analytical balance Mettler Toledo X according to ISO 17304:2013 (E).

In order to measure the density of the unpolymerized sample, a glass dish was used. Firstly, the mass of the glass dish was weighed in air and in water, and the density of the glass dish ρ_gd_ was calculated according to Equation (2):

(2) $$\rho_{\text{gd}}=\frac{m_{\text{gd}1}\times \rho_{0}}{m_{\text{gd}1}-m_{\text{gd}2}}$$

where ρ_0_ is the density of water, *m*_gd1_ and *m*_gd2_ are the mass of the glass dish in air and in water, respectively.

The measurement of the density of the glass dish was repeated three times to get the mean value ρ_gd,m_.

Then, a certain amount of the unpolymerized sample was dispensed into the glass dish, the total mass of the sample and the glass dish was weighed in air and in water, and the density of the unpolymerized sample ρ_up_ was calculated according to Equation (3)

(3) $$\rho_{\text{up}}=\frac{m_{\text{ud}1}-m_{\text{gd}1}}{m_{\text{ud}1}-\left(\frac{m_{\text{gd}1}\times \rho_{0}}{\rho_{\text{gd},m}}\right)-m_{\text{ud}2}}\times \rho_{0}$$

where *m*_gd1_ is the mass of the glass dish in air, *m*_ud1_ is the mass of the unpolymerized sample and the glass dish measured together in air, m_ud2_ is the mass of the unpolymerized sample and the glass dish measured together in water, ρ_gd,m_ is the mean density of the glass dish, ρ_0_ is the density of water.

After the measurement, the glass dish was cleaned carefully, and the measurement was repeated on four more samples to get the mean value of the density of the unpolymerized sample ρ_up,m_.

Resins were poured into a steel mold sized 2 mm × 2 mm × 25 mm, then light-cured using the same dental light source as shown in DC measurement (60 s for one portion until the whole sample was irradiated). The mass of the polymerized sample (size) was weighed in air and in water, and the density of the polymerized sample ρ_ps_ was calculated according to Equation (4)

(4) $$\rho_{\text{ps}}=\frac{m_{\text{ps}1}\times \rho_{0}}{m_{\text{ps}1}-m_{\text{ps}2}}$$

where ρ_0_ is the density of water, *m*_ps1_ is the mass of the polymerized sample in air, *m*_ps2_ is the mass of the polymerized sample in water.

The measurement on the polymerized sample was repeated five times to get the mean value of the density of the polymerized sample ρ_ps,m_.

Finally, the VS of the sample was calculated according to Equation (5)

(5) $$\text{VS}=\frac{\rho_{\text{ps},m}-\rho_{\text{up},m}}{\rho_{\text{ps},m}}\times 100\%$$

### 4.7. Measurement of Water Sorption and Solubility

Resins were poured into a cylindrical steel mold (15 mm in internal diameter and 1.00 mm in height) between two transparent Mylar sheets, and a glass slide with 1 mm thickness was used to cover the upper surface. After that, the samples were irradiated (60 s for one portion until the whole sample was irradiated) using the same dental light source as shown in DC measurement. For each dental resin system, five specimens were prepared. The initial weight (M_1_) of every specimen was measured with an electronic balance (Mettler A30, Mettler Instrument Co., Highstone, NJ, USA) with an accuracy of 0.1 mg. Then, the specimens were put into 30 mL of distilled water and stored at 37 °C. At fixed time intervals, they were removed, blotted dry to remove excess water, re-weighed and returned to the water. At 30 days of immersion, there was no significant variance in mass and M_2_ was obtained as equilibrium mass. The specimens were then dried at 60 °C until their mass kept constant, and the result was recorded as M_3_. Water sorption and solubility were then calculated using Equations (6) and (7):

(6) $$\text{WS}=\frac{M_{2}-M_{3}}{V}\times 100\%$$

(7) $$\text{WSL}=\frac{M_{1}-M_{3}}{V}\times 100\%$$

### 4.8. Measurement of Flexural Strength and Modulus

Resins were poured into a steel mold sized 2 mm × 2 mm × 25 mm, then light-cured using the same dental light source as shown in DC measurement (60 s for one portion until the whole sample was irradiated). Eight specimens were prepared for every dental resin system. Flexural strength (FS) and modulus (FM) were investigated by a three-point bending test (span, 20 mm) according to ISO 10477:92 standard with a universal testing machine (Model Z010, Zwick GmbH & Co. KG, Ulm, Germany), at a cross-head speed of 1.00 mm/min. The FS in MPa was then calculated using Equation (8):

(8) $$\text{FS}=\frac{3\;pL}{{2\;\text{bd}}^{2}}$$

where *p* stands for load at fracture (N), *L* is the span length (20 mm), and b and d are the width and thickness of the specimens in mm, respectively. The FM was also determined from the slope of the initial linear part of the stress-strain curve.

Another eight samples of each resin formulation were prepared and immersed in water for 30 days. The FS and FM of samples after water immersion were also investigated.

### 4.9. Statistical Analysis

The results of viscosity measurement were subjected to the Student’s *t*-test. All the other results were statistically analyzed and compared using one-way ANOVA and Tukey’s test at the significance level of 0.05 by software SPSS 13.0 (SPSS Inc., Chicago, CA, USA).

## 5. Conclusions

Despite the limitations of this work, it could be concluded that the synthesized fluorinated dimethacrylate FDMA had potential to be used as a substitute for Bis-GMA in dental resin because FDMA-based resin had several advantages when compared with Bis-GMA-based resin, such as higher double bond conversion, lower volumetric shrinkage, and better water resistance. Though water solubility of FDMA-based resin was a little bit higher, more research is still needed, such as biocompatibility testing, to prove whether it would influence FDMA-based resin used in clinic. Moreover, further research should also be undertaken to investigate whether FDMA-based material had resistance to an oral microbial attachment.

## Figures and Tables

**Table 1:** Viscosity, double bond conversion at an irradiation time of 60 s (DC60s) and volumetric shrinkage (VS) of each resin system.

Resin Formulation	Viscosity (mPa·s)	DC60s (%)	VS (%)
Bis-GMA/TEGDMA	164.7 ± 1.5 ^A^	53.8 ± 2.1 ^a^	9.2 ± 0.8 ^a^
FDMA/TEGDMA	201.7 ± 1.5 ^B^	65.4 ± 3.7 ^b^	7.8 ± 0.9 ^b^

^A,B^ Different upper case letters indicate statistical differences with a column by the Student’s *t*-test (*p* < 0.004); ^a,b^ Different lower case letters indicate statistical differences with a column (*p* < 0.05).

**Table 2:** Flexural strength (FS), flexural modulus (FM), water sorption (WS), and solubility (WSL) of cured resins.

Resin Formulation	FS (MPa)		FM (GPa)		WS (%)	WSL (%)
	Before Water Immersion	After Water Immersion	Before Water Immersion	After Water Immersion		
Bis-GMA/TEGDMA	100.3 ± 6.0 ^a,A^	77.6 ± 5.3 ^a,B^	2.38 ± 0.05 ^a,C^	1.83 ± 0.10 ^a,D^	4.08 ± 0.05 ^a^	0.83 ± 0.08 ^a^
FDMA/TEGDMA	96.5 ± 4.6 ^a,A^	88.6 ± 4.5 ^b,B^	2.33 ± 0.08 ^a,C^	1.99 ± 0.13 ^a,D^	3.85 ± 0.04 ^b^	1.14 ± 0.09 ^b^

^a,b^ Different lower case letters indicate statistical differences with a column (*p* < 0.05); ^A,B,C,D^ Different upper case letters indicate statistical differences between FSs or FMs of the same cured resin before and after immersion (*p* < 0.05).
